# Social determinants of vulnerability to nitrogen oxide- and sulfur dioxide-related bone damage among postmenopausal women in the United States

**DOI:** 10.3389/fpubh.2026.1832667

**Published:** 2026-06-30

**Authors:** Diddier Prada, Andrea Ramírez, Aravind Lathika Rajendrakumar, Andrew Maroko, James D. Stewart, Duanping Liao, David Cantu-de-León, Claudia García-Cuellar, Jonathan González-Ruíz, Eric A. Whitsel, Andrea A. Baccarelli, Carolyn J. Crandall, Haritz Irizar, Mayte Suárez-Fariñas, Douglas Kiel, Shivani Sahni, Carol R. Horowitz

**Affiliations:** 1 Institute for Health Equity Research, Icahn School of Medicine at Mount Sinai, New York, NY, United States; 2 Department of Population Health Science and Policy, Icahn School of Medicine at Mount Sinai, New York, NY, United States; 3 Department of Environmental Medicine, Icahn School of Medicine at Mount Sinai, New York, NY, United States; 4 Subdirección de Investigación Básica, Instituto Nacional de Cancerología, Tlalpan, Ciudad de Mexico, Mexico; 5 Department of Epidemiology, Gillings School of Global Public Health, University of North Carolina, Chapel Hill, NC, United States; 6 Department of Public Health Science, Pennsylvania State University College of Medicine, Hershey, PA, United States; 7 Department of Medicine, School of Medicine, University of North Carolina, Chapel Hill, NC, United States; 8 Harvard T. H. Chan School of Public Health, Harvard University, Boston, MA, United States; 9 Division of General Internal Medicine and Health Service Research, David Geffen School of Medicine at the University of California, Los Angeles, Los Angeles, CA, United States; 10 Department of Medicine, Hinda and Arthur Marcus Institute for Aging Research, Hebrew SeniorLife, Beth Israel Medical Center and Harvard Medical School, Boston, MA, United States

**Keywords:** bone mineraI density, nitrogen oxide (NO) x, postmenopausal women, social determinants of health, sulfur dioxide

## Abstract

**Background:**

Studying social determinants of health (SDOH) in air pollutant-related bone loss among aging women may help identify bone health disparities, design preventive strategies, and inform policy. Prolonged exposure to air pollution is associated with decreased bone mineral density (BMD) and increased fracture risk in later life. However, the extent to which socioeconomic status (SES) contributes to pollution-related bone damage remains unclear.

**Methods:**

We estimated the extent to which social determinants of health (SDOH) modify the associations of nitrogen oxides (NOx) and sulfur dioxide (SO_2_)-induced bone damage. We analyzed the Women's Health Initiative (WHI) data from the randomized clinical trial (RCT) (*n* = 4,202) and the observational study (OS) (*n* = 4,839), totaling 9,041 women. Ambient concentrations of nitrogen dioxide (NO_2_), nitrogen oxide (NO), and SO_2_, averaged over 1-, 3-, and 5-year periods preceding bone mineral density (BMD) assessment, were the exposures. Individual- and neighborhood-level SDOH (self-reported educational attainment, annual family income, and neighborhood-level socioeconomic status (nSES) were evaluated as potential effect modifiers. BMD at the lumbar spine, total hip, and whole body were modeled as outcomes using linear mixed models.

**Results:**

A 10% increase in NO_2_ was suggestively associated with 1.4–3.8 times greater BMD loss in low vs. high nSES participants across all sites. For example, 1-year lumbar spine BMD loss per 10% NO_2_ increase was β = −0.047 (95% CI, −0.066 to −0.027) in the low nSES compared with β = −0.013 (95% CI, −0.023 to −0.001) in the high nSES group (*p*-interaction = 0.001). Similarly, suggestive associations were also observed for NO. nSES interactions were null for SO_2_. For income, NO was suggestively associated with lumbar spine bone loss across all exposure periods, whereas only the 3-year average NO_2_ exposure was suggestively associated with whole-body BMD (*p* = 0.048). Again, no interaction was observed for SO_2_. Participants with lower education showed greater NO-related lumbar spine bone. SO_2_ showed the opposite pattern at multiple sites, while NO_2_ showed no associations.

**Conclusion:**

Neighborhood-level analyses revealed suggestive associations between bone mineral loss and a broader range of air pollutants. Reducing social inequities may improve the bone health of older women living in areas with high air pollution. Additional studies are needed to generalize the findings to younger women and men, as only older women were analyzed.

## 1 Introduction

Osteoporosis is a bone disorder that reduces bone strength and mineral density, thereby increasing the risk of fractures (1). The burden of osteoporosis-related fractures is high in the United States (U.S.), with around 2.1 million cases reported (2), resulting in $22 billion annual direct healthcare costs (3). Individuals with osteoporosis experience decreased quality of life (QOL), comorbidities, and even higher mortalities (4). Postmenopausal women are more vulnerable, with one in two women over the age of 50 years likely to experience a bone fracture due to osteoporosis (5, 6). Social determinants of health (SDOH) refer to the conditions in which people are born, grow, live, work, play, and age, including access to healthcare (7). These factors can influence a wide range of health risks and outcomes (8). Financial strain, low levels of educational attainment, or unstable employment can increase the risk for chronic diseases (9), including mental health issues (10), and also negatively affect functional abilities (11). Multiple conceptual frameworks of SDOH exist that aim to link it to health inequalities (12). One of the most widely recognized models is the World Health Organization (WHO) model, whereas competing perspectives have questioned whether SDOH effects can be explained in biological terms (13).

Social determinants of health (SDOH) shape a wide range of health risks and outcomes at both the group and individual levels. At the neighborhood level, it determines access to food, housing quality, education opportunities, population density, and other geographic characteristics—collectively referred to as neighborhood-level socioeconomic status (nSES) (14, 15). Adverse SDOH factors at the individual level can accelerate the progression of age-related conditions such as cardiovascular disease (16), diabetes (17), and osteoporosis (18). Simultaneously, they limit individuals' access to quality healthcare, nutritious food, safe housing, and supportive social networks (19) — factors critical for maintaining health and improving outcomes in older age.

Marginalized communities experience an unusual confluence of risk factors that is not observed in the general population (20). For instance, they are exposed to higher levels of environmental toxicants as they are more likely to live near primary pollution sources, such as industries and landfills, which generate high levels of air pollution (21). Toxic constituents of air pollution are important risk factors for several diseases, including osteoporosis, and severely impair cellular functioning and metabolism (22, 23). Loss of bone density caused by air pollution was even observed in young individuals in a South Indian study (24). Causal analysis in middle-aged individuals has further strengthened the evidence for bone loss, particularly for nitrogen oxides (NO_x_) (25). However, a meta-analysis has found no significant associations, (26) and specific contaminants, such as SO_2_, have usually received less attention in studies (27–29). Participant characteristics, such as socioeconomic status and lived experiences, have not been thoroughly investigated beyond inclusion as model covariates to control for confounding (26). Therefore, in this study, we evaluate whether the association between cumulative exposure to NO_x_ and SO_2_ and bone density is synergistically modified by individual- and neighborhood-level social determinants among postmenopausal women in the U.S. We hypothesized that low nSES would amplify NO_x_ and SO_2_ associations with BMD more than individual SDOH.

## 2 Subjects and methods

### 2.1 Study population and design

The Women's Health Initiative (WHI) includes detailed data on 161,808 postmenopausal women aged 50–79 enrolled at 40 clinical centers across the U.S. between October 1993 and December 1998. Written informed consent was obtained from all participants at the time of randomization or enrollment, following Institutional Review Board approval at each WHI clinical center site. Women were enrolled in one or more of three overlapping randomized clinical trials (RCT; *N* = 68,132) or an observational study (OS; *N* = 93,676). The trials tested menopausal hormone therapies, including estrogen plus progestin (E+P) vs. placebo in women with an intact uterus (*n* = 16,608) and estrogen alone (E-alone) vs. placebo in women with a prior hysterectomy (*n* = 10,739), dietary modification, calcium/vitamin D supplementation, and hormone therapy. A WHI BMD substudy, measuring BMD at enrollment and at 1, 3, 6, and 9 years of follow-up, included all participants from three clinical centers and one satellite clinic (*N* = 11,020). After excluding participants without air pollution estimates, the sample analyzed included 4,202 RCT participants and 4,839 OS participants (total *N* = 9,041) with BMD and air pollution data at enrollment.

### 2.2 Air pollution exposure assessment

First, we accurately geocoded the addresses of WHI participants from the cohort's inception in 1993 through 2018 (30). We then computed participant address-specific daily mean concentrations of air pollutants—nitric oxide (NO), nitrogen dioxide (NO_2_), and SO_2_–from 1993 to 2012. These estimates were derived from the U.S. Environmental Protection Agency (EPA) Air Quality System data using national-scale log-normal ordinary kriging (30). Model validity was assessed by evaluating the proximity of the mean prediction error (PE) and standardized prediction error (SPE) to zero, the standardized root mean square (RMSS) to one, and the root mean square (RMS) to the standard error (SE) (31).

### 2.3 Individual and neighborhood-level SDOH data

Individual-level SDOH were self-reported at baseline. For this study, we used self-reported educational attainment (categories collected: did not go to school, grade school, some high school, high school, high school/trade school/GED, some college or an associate degree, bachelor's degree or higher) and annual family income (categories collected: < $20,000, $20,000-$34,999, $35,000-$49,999 vs. $50,000-$74,999, ≥$75,000/year). Low education and low income were defined as a high school education or less, and an annual income of ≤ $50,000, respectively.

Neighborhood-level socioeconomic status (nSES) was assessed using a composite index derived from six U.S. Census tract-level variables: (1) median household income; (2) median value of housing units; (3) percentage of households receiving interest, dividend, or rental income; (4) percentage of adults aged ≥25 years who completed high school; (5) percentage of adults aged ≥25 years who completed college; and (6) percentage of employed individuals aged ≥16 years in executive, managerial, or professional occupations. Each variable was standardized (z-transformed), and the sum of these values constituted the nSES score, expressed in standard deviation (SD) units. The nSES score was dichotomized at the median into low and high nSES categories, with higher scores indicating residence in more socioeconomically advantaged neighborhoods (15).

Ambient concentrations of NO_2_, NO, and SO_2_ averaged over 1-, 3-, and 5-year periods preceding BMD assessment were the primary exposures. Individual- and neighborhood-level SDOH (self-reported educational attainment, annual family income, and neighborhood-level socioeconomic status (nSES) were evaluated as potential effect modifiers.

### 2.4 Outcome assessment

The main outcomes of the study were BMD at the lumbar spine, total hip, and whole body. Dual-energy X-ray absorptiometry (DXA) scans were performed using Hologic machines (QDR 2000, 2000+, or 4500), with quality assurance procedures that included cross-clinic calibration using phantoms and periodic review of randomly selected scans. When machines were upgraded from QDR 2000 to QDR 4500, *in vivo* cross-calibration was performed, and correction factors were applied to account for longitudinal changes. Certified technicians measured BMD in g/cm^2^ at the total hip, lumbar spine, and total body using standardized protocols during screening and annual visits. WHI quality assurance procedures included the routine use of spine and hip phantoms, with interscanner variability maintained at < 1.5% for the spine, < 4.8% for the hip, and < 1.7% for linearity (23).

### 2.5 Statistical analysis

All statistical analyses were conducted using R (version 4.3.3; R Core Team, 2024). We examined the distribution of all variables and identified potential outliers through visual inspection using quantile–quantile (Q–Q) plots (qqnorm function in R), and formal testing using the Kolmogorov–Smirnov test (ks.test) for normality and Grubbs' test (grubbs.test) for outliers. Continuous variables with non-normal distributions were log-transformed to improve normality. Missing data, which accounted for less than 5% across all variables, were addressed using multiple imputation via the *mice* package, with five imputations performed per analysis (32, 33). We initially examined correlations between pairs of air pollutants using the Pearson product-moment correlation coefficient (r), after confirming normality for each variable. Longitudinal associations between air pollutant concentrations and bone mineral density (BMD) across study visits were estimated using generalized linear mixed models (GLMMs):

*y*_ij =_ β_0_ + β_1_*Concentration*_i*j*_ + β_2_*SDOH*_i*j*_ + β_3_*x*_i*j*_ + *u*_j_ + ε_ij_,

where y_ij_ is the BMD at the *i*^th^ observation of the *j*^th^ participant, β_0_ is the fixed intercept, β_1_*-*β_3_ are fixed coefficients (β_1_ for a given air pollutant concentration, β_2_ for a given SDOH, and β_3_ for a vector of participant characteristics *x*_i*j*1_,…, *x*_i*jn*_), *u*_j_ is the random intercept for the *j*^th^ participant, and ϵ_ij_ is the residual error for the *i*^th^ observation in the *j*^th^ participant.

In this model, nSES was dichotomized at its median ( ≤ -2·56), income at ≤ $50,000/year, and education at ≤ high school. All models were adjusted for key covariates, including demographic characteristics [age (in years) at the time of BMD assessment, race/ethnicity, US Census region, and clinical center—both region and center defined at randomization/enrollment]; behavioral characteristics [smoking status (never vs. former/current) and physical activity (measured in MET-hours/week)]; and clinical characteristics [study membership (Observational Study vs. Randomized Controlled Trial), randomization arms (hormone therapy vs. placebo, low-fat diet vs. control, and calcium/vitamin D vs. placebo), and body mass index (kg/m^2^)].

Linear mixed models were used to examine the associations between selected air pollutants and longitudinal BMD measurements. To evaluate the potential effect modification of the NO_x_ and SO_2_-BMD associations by baseline SDOH, interaction terms between each air pollutant and each SDOH variable were included in the models containing the corresponding main effects.

## 3 Results

### 3.1 Participant characteristics

On average, WHI participants were 63.3 years old at baseline (standard deviation [SD]: 7.2 years) (Table 1). Most participants were White, and the most common level of educational attainment was college or vocational training. The majority had an annual income of less than $50,000, were never smokers, and reported low levels of physical activity.

**Table 1:** Demographic characteristics of women in the WHI at enrollment (*N* = 9,041) with available bone mineral density and long-term NO_x_ and SO_2_ data.

Variable	Mean/n	SD/%
**Age**	63.3	7.2
**Ethnicity/Race**		
White (non-Hispanic)	7,068	78.2%
Black	1,229	13.6%
Hispanic	552	6.1%
Others	175	1.9%
Not available	17	0.2%
**Income level**		
Lower	4,532	50.1%
Higher	3,882	42.9%
Not available	627	6.9%
**Education level**		
Lower	3,833	42.49%
Higher	5,148	56.9%
Not available	60	0.7%
**nSES**		
Lower	2,379	26.3%
Higher	6,662	73.7%
**Alcohol consumption**		
Never	1,507	16.7%
Former	1,927	21.3%
< 7 drinks per week	4,826	53.4%
≥7 drinks per week	713	7.9%
Not available	68	0.7%
**Smoking**		
Never	4,988	55.2%
Former	3,255	36.0%
Current	688	7.6%
Not available	110	1.2%
**Physical activity, MET-hours/week**	0.0407	0.20
**Body mass index, kg/m2**		
≤ 18.5	79	0.9%
18.6–24.9	2,745	30.4%
≥25 kg/m2	6,187	69.4%
Not available	30	0.3%
**Bone mineral density (corrected), g/cm** ^**2**^		
Total Hip	0.8547	0.1
Lumbar spine	0.9804	0.2
Whole body	1.0129	0.1
**Region**		
Northeast	3,067	33.9%
South	3,055	33.8%
West	2,919	32.3%
**Clinical trial components (HRT, CAD, DM)**		
Yes	4,202	46.5%
No	4,839	53.5%
**Dietary modification study arm**		
Yes	3,059	33.8%
No	5,982	66.2%
**Calcium and vitamin D study arm**		
Yes	2,272	25.1%
No	6,769	74.9%
**Hormone replacement therapy study arm**		
Yes	1,642	18.2%
No	7,399	81.8%

Air pollutant concentrations were correlated weakly for NO and SO_2_ (*r* = 0.08–0.11), more strongly for NO_2_ and SO_2_ (*r* = 0.34–0.40), and very strongly for NO_2_ and NO (*r* = 0.85–0.86) across the 1-, 3-, and 5-year averaging periods (Supplementary Table S1).

### 3.2 SDOH-stratified estimates of No_*x*_ and SO_2_ associations with BMD

**Figure 1:**
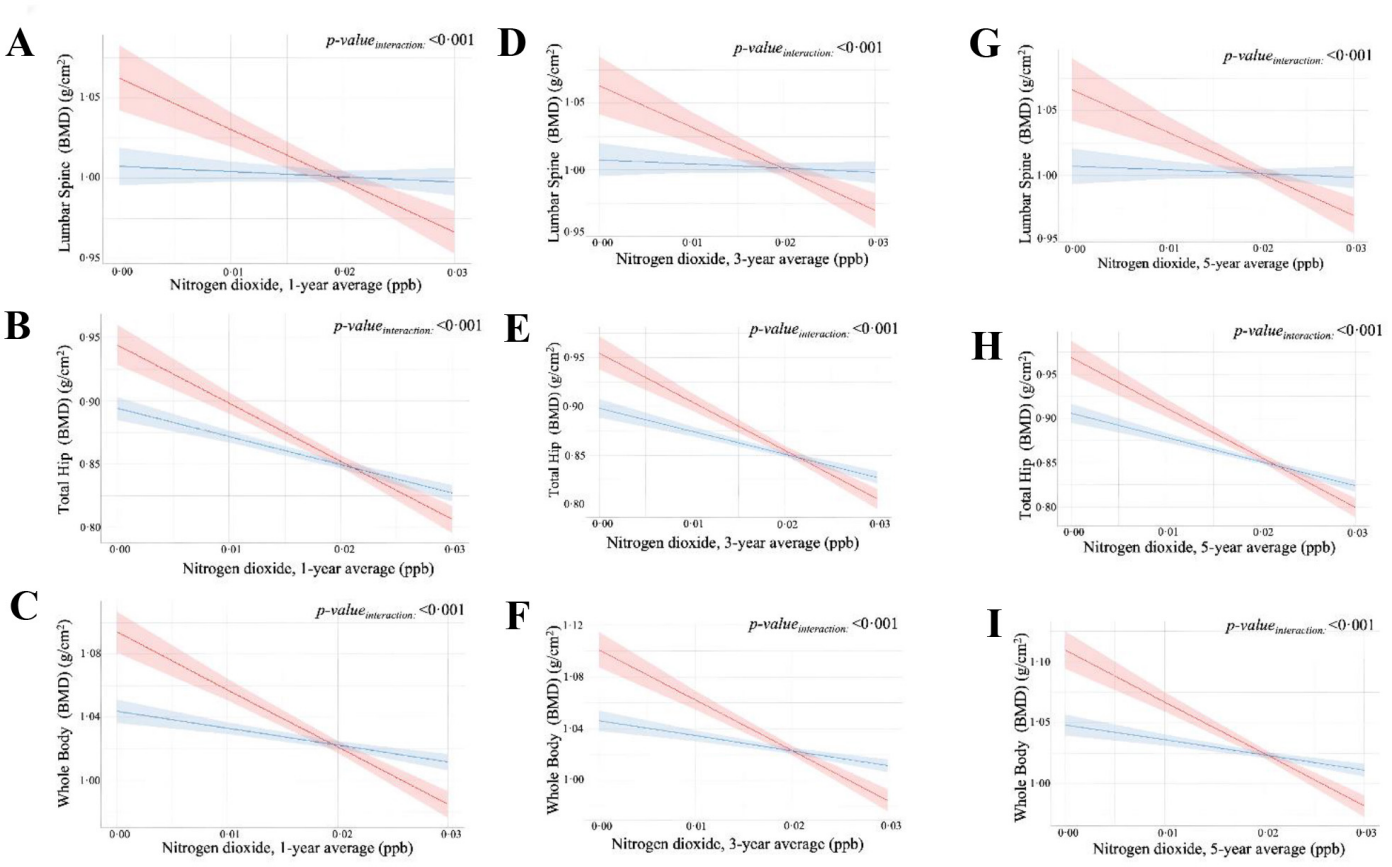
Effect modification of neighborhood socioeconomic status (nSES) in the association between 1-, 3-year average and 5-year average of NO_2_ on BMD in postmenopausal women. **(A–C):** 1-year average **(A)**. Lumbar spine BMD. **(B)**. Total Hip BMD. **(C)**. Whole-body BMD). **(D–F):** 3-year average **(D)**. Lumbar spine BMD. **(E)**. Total Hip BMD. **(F)**. Whole-body BMD). **(G–I)**: 5-year average **(G)**. Lumbar spine BMD. **(H)**. Total Hip BMD. **(I)**. Whole-body BMD). Predicted values (lines) with 95% confidence intervals (shaded areas) shown for low (red) and high (blue) nSES.

**Figure 2:**
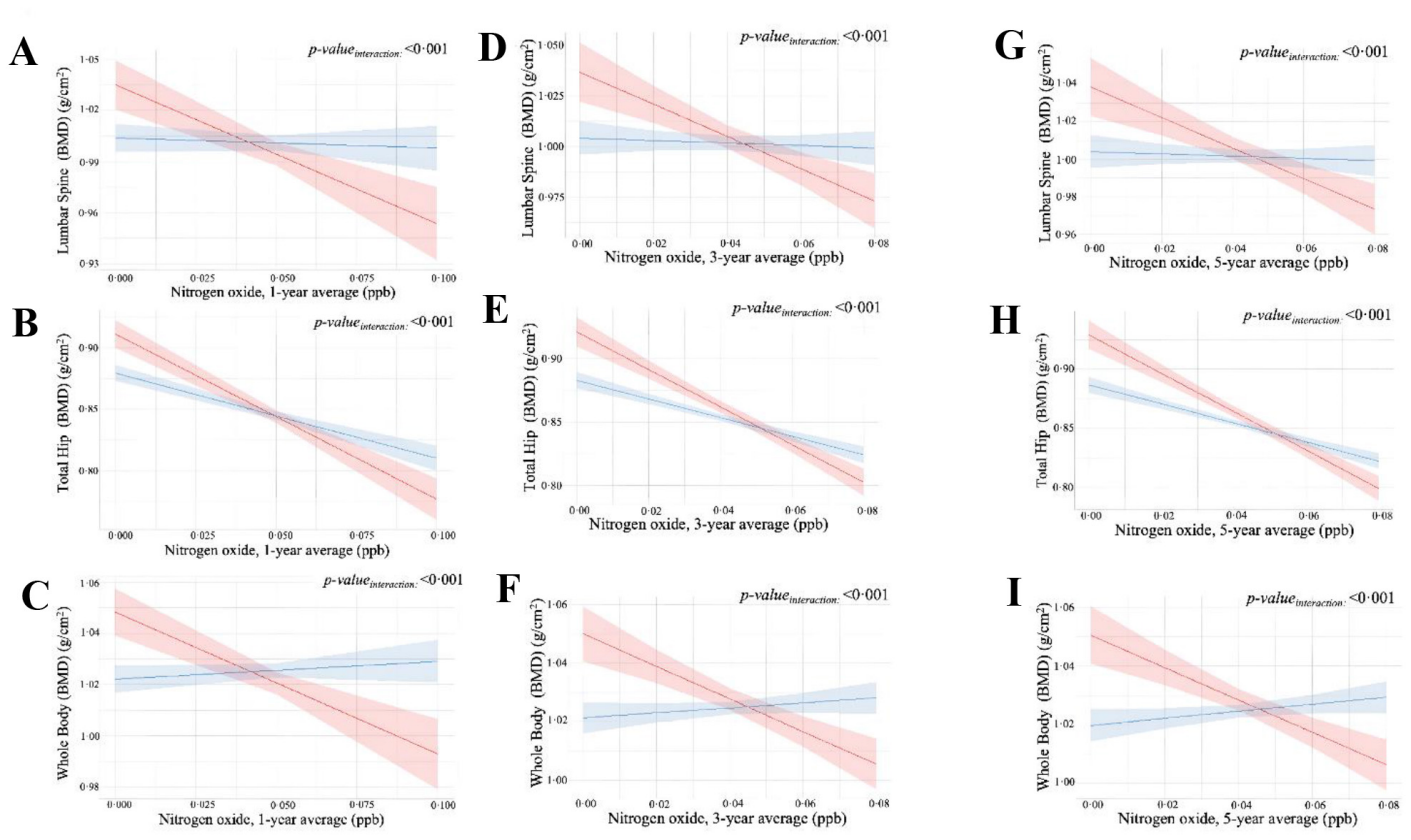
Effect modification of neighborhood socioeconomic status (nSES) in the association between 1-, 3-year average, and 5-year average of NO on BMD in postmenopausal women. **(A–C):** 1-year average **(A)**. Lumbar spine BMD. **(B)**. Total Hip BMD. **(C)**. Whole-body BMD). **(D–F):** 3-year average **(D)**. Lumbar spine BMD. **(E)**. Total Hip BMD. **(F)**. Whole-body BMD). **(G–I)**: 5-year average **(G)**. Lumbar spine BMD. **(H)**. Total Hip BMD. **(I)**. Whole-body BMD). Predicted values (lines) with 95% confidence intervals (shaded areas) shown for low (red) and high (blue) nSES.

**Figure 3:**
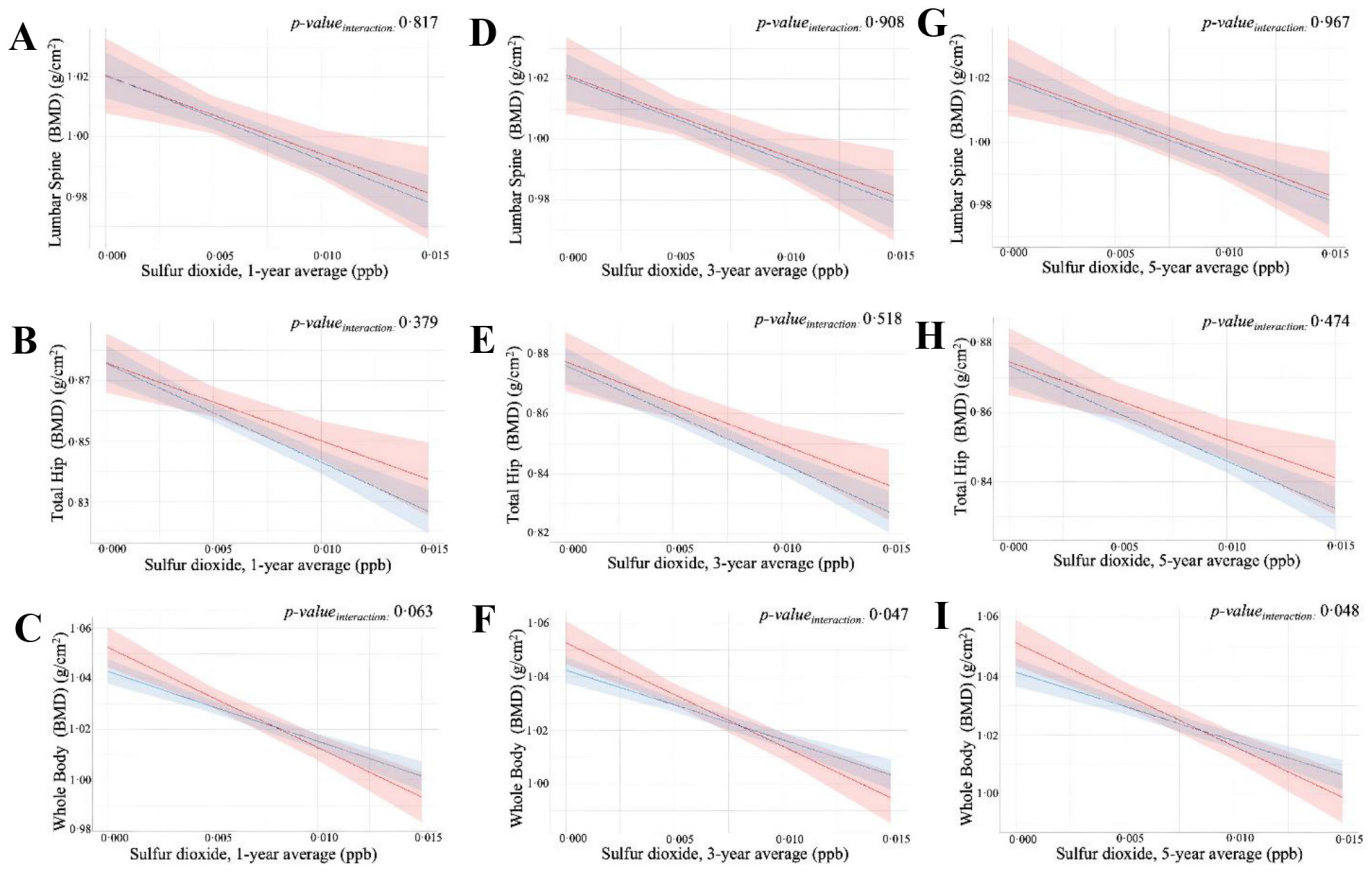
Effect modification of neighborhood socioeconomic status (nSES) in the association between 1-, 3-, and−5-year average of SO_2_ on BMD in postmenopausal women. **(A–C):** 1-year average **(A)**. Lumbar spine BMD. **(B)**. Total Hip BMD. **(C)**. Whole-body BMD). **(D–F):** 3-year average **(D)**. Lumbar spine BMD. **(E)**. Total Hip BMD. **(F)**. Whole-body BMD). **(G–I)**: 5-year average **(G)**. Lumbar spine BMD. **(H)**. Total Hip BMD. **(I)**. Whole-body BMD). Predicted values (lines) with 95% confidence intervals (shaded areas) shown for low (red) and high (blue) nSES.

We evaluated the modification of the air pollutant-BMD association by baseline individual- and neighborhood-level SDOH across three averaging periods (i.e., 1-, 3-, and 5-year) at three BMD sites (lumbar spine, hip, whole-body). Figures 1–3 depict the relationship between ambient air pollutants and bone loss by anatomical site and duration of exposure, stratified by low vs. high nSES. At the neighborhood level, as shown in Table S2, we found that for lumbar spine BMD and 1-year average NO_2_ exposure, postmenopausal women living in neighborhoods with low nSES experienced markedly greater bone mineral loss [β = −0.047, 95% Confidence Interval (CI): −0.066, −0.027 per 10% increase) compared with those residing in high nSES neighborhoods (β = −0.013, 95% CI: −0.023, −0.001; *p*-interaction = 0.001).

For total hip BMD, women living in low nSES neighborhoods had 1.6 times larger coefficients (β = −0.067, 95% CI: −0.135, −0.001 per 10% increase) compared to those in high nSES neighborhoods (β = −0.042, 95% CI: −0.049, −0.033; p-interaction = 0.001). Similarly, for whole-body BMD, women in low nSES neighborhoods had 2.3 times larger coefficients (β = −0.072, 95% CI: −0.073, −0.071) than those in high nSES neighborhoods (β = −0.031, 95% CI: −0.037, −0.024; p-interaction = 0.001). Comparable trends were observed for 3- and 5-year mean NO_2_ exposures. At the neighborhood level, for 1-year average NO exposure and lumbar spine BMD, postmenopausal women living in neighborhoods with the lowest nSES showed effect estimates showed a 2-fold increased bone loss [β = −0.027, 95% Confidence Interval (CI): −0.041, −0.014] for low nSES neighborhoods than those living in higher nSES neighborhoods (*p*-interaction = 0.002) (*p*-interaction = 0.002). For total hip BMD and 1-year NO exposure, women in low nSES neighborhoods had 1.5 times higher effect estimates (β = −0.043, 95% CI: −0.052, −0.033) than those in high nSES neighborhoods (β = −0.029, 95% CI: −0.035, −0.024; *p*-interaction = 0.001). For whole-body BMD, the effect estimates were 3 times larger among women in low nSES neighborhoods (β = −0.033, 95% CI: −0.042, −0.020) compared with those in high nSES neighborhoods (β = −0.011, 95% CI: −0.016, −0.006; p-interaction = 0.001). Similar trends were observed for 3- and 5-year average NO exposures.

For SO_2_ and nSES, there were no interactions at any of the evaluated time windows. Estimates, 95% confidence intervals (CIs), and p-values for interaction across 1-, 3-, and 5-year averages are presented in Supplementary Table S2. At the individual level, we found that for lumbar spine BMD and 1-year average NO exposure, women with the lowest income had effect estimates that were twice as large as those for women with higher income (low income: β = −0.022, 95% CI: −0.032, −0.012; high income: β = −0.011, 95% CI: −0.013, −0.010; *p*-interaction = 0.025).

No significant differences were observed between income groups for NO_2_, except for whole-body BMD, where the association was limited to 3-year average NO_2_ exposure and borderline significant (Supplementary Table S3). Differences across income levels for NO were significant for lumbar spine regardless of exposure duration. However, no interactions with income were observed for SO_2_.

Supplementary Table S4 presents detailed analyses of effect modification by education. No interaction with education was observed for NO_2_ across any of the time windows evaluated. Still, we found associations between NO and SO_2_ and lumbar spine and total hip BMD at 1-, 3-, and 5-year averages. For lumbar spine BMD and the 1-year average NO exposure, postmenopausal women with the lowest education had larger effect estimates than for women with high education (low education, β: −0.017, 95% CI: −0.027, −0.007; high education, β: −0.012, 95% CI: −0.020, −0.004; p-interaction: 0.008). Similar trends were observed at 3- and 5-year average NO exposure.

For lumbar spine and total body BMD at 1-, 3-, and 5-year averages, SO_2_ exposure had stronger associations among women with higher education. At the lumber spine BMD: low education, β: −0.013, 95% CI: −0.023, −0.003; high education: β: −0.017, 95% CI: −0.027, −0.007; *p*-interaction: 0.024. Similar trends were observed at 3- and 5-year average SO_2_ exposure. For total body BMD: low education, β: −0.030, 95% CI: −0.038, −0.022; high education: β: −0.040, 95% CI: −0.046, −0.034; *p*-interaction: 0.005). Similar trends were observed at 3- and 5-year average SO_2_ exposures.

## 4 Discussion

Our study suggests that women living in marginalized localities are more vulnerable to bone loss in response to NO_x_ exposures. Depending on the adverse SDOH measures explored, disadvantaged individuals in our data were 1.5 to times more likely to experience greater bone loss than those better off socioeconomically. Risk factors of disease tend to cluster in underprivileged communities, (34) which might considerably reduce resilience against the harmful effects of NO_x_ (35). Research across Asia (24, 36–41), the U.S., (23) and the United Kingdom (42) have consistently shown that NOx and SO_2_ drive bone mineral loss and fractures. However, examining air pollution and its interactions poses generalizability challenges due to substantial differences in population characteristics and health systems (43).

Our earlier study reported greater risk of fracture hospitalizations in U.S. communities in the lowest vs. the highest income quartiles with high particulate matter levels, (44) suggesting that poverty is a risk factor for air pollution-related bone damage. Subsequent research indicated that NO_x_, rather than particulate matter, was a stronger predictor of bone density changes and time points (23). However, bone loss in the total hip, femoral neck, and lumbar spine was greater with SO_2_ exposure than with particulate matter or NO_x_ (23). In the present study, of all the bone outcomes examined with NO_x_ and SO_2_ exposures, total hip BMD generally showed the most pronounced decline over time. The bone mineral loss patterns for different pollutants were same across the different nSES groups demonstrating a better ability to delineate the associations arising due to social adversity. In comparison, greater bone mineral loss was observed for NO_2_ in the high-income category with early exposure (1-year average). Such an increase was again observed for all the pollutants, especially SO_2_. Bone loss was also observed in the higher-income group at other exposure durations for NO_2_ and SO_2_. However, this finding is not without precedent. Considerable heterogeneity has been reported across studies in the relationship between osteoporotic bone health and income or education (45). In our study, heterogeneity was more apparent in education strata. A Danish study reported that higher education transitioned from a protective factor to a risk factor for fractures in older age (46). Therefore, the bone outcome associations may vary across educational strata depending on other risk factors. Another view is that education does not exert direct effects on bone mass, but rather acts indirectly through height and body mass, which prevents finding associations even if they exist when exploring bone mass or fractures (47). Still, we found that having both levels of SDOH into statistical models improves the precision of estimates of environmentally related damage.

Previous studies have not explicitly investigated the combined effects of SDOH and NO_x_ and SO_2_ on bone health (23, 40, 48). Our own study of bone loss in the Medicare data among lower-income individuals was limited to particulate matter exposure (44). Vehicular emissions are the main source of NO_x_ (49). The biological mechanisms that link social adversity to NO_x_-related bone loss are not fully understood. Osteoporosis risk was lower in participants in the Health and Retirement Study (HRS) with better socioeconomic status (50). First and foremost, individuals from deprived neighborhoods tend to have poorer overall health than those from wealthier areas, increasing their susceptibility to air pollution (51). Social adversity and air pollution affect health not merely through the downstream processes but also intersect at genetic and other molecular levels (52, 53). Criteria pollutants like NO_x_ interfere with cell signaling, impair mitochondrial activity, and suppress DNA methylation (54, 55). DNA methylation patterns in black individuals have revealed that the combination of social deprivation and air pollution accelerated biological aging at nearly twice the expected rate (56). According to this study, neighborhood variables were more informative in predicting biological age than education and wealth (56). Another epigenetically mediated mechanism that may contribute to bone mineral loss is inflammation, which is commonly elevated in individuals exposed to air pollutants and social adversity (53, 55). High levels of inflammation affect bone integrity by stimulating osteoclastogenesis and increasing bone demineralization (40, 57). Collective evidence suggests that heightened physiological stress promotes oxidative reactions, induces apoptosis in bone cells, and alters pathways such as glucocorticoid signaling (58). In agreement, a study investigating the influence of income and race found increased bone remodeling in response to elevated social stress (59). Therefore, it is highly likely that chronic exposure to both NO_x_ and social adversity may synergistically elevate the risk of age-related conditions beyond the risk posed by either factor alone. Both individual- and population-level measures of SDOH capture distinct dimensions of social adversity and should therefore be investigated accordingly, as demonstrated in prior research (60). One possible reason for the better performance of nSES may be that it also includes several components of the individual-level social determinants, such as housing value. Alternatively, the nSES index integrates multiple housing-related indicators that reflect not only the built environment but also wider community characteristics and social vulnerabilities.

It is no overstatement to say that osteoporosis is a predominantly female-centered disease, as only about 20% of those affected are men (61). Income inequality in the U.S. has reportedly increased over the last few decades (62). Intergenerational mobility in the U.S. has declined over time, with growing correlation in occupational status between parents and children, highlighting the persistence of social disadvantage (63). A recent study reported that awareness about the harmful effects of air pollution among the U.S. population is lower than desired (below 50%) (64). At the same time, according to the latest statistics from the American Lung Association, it is estimated that close to half of the U.S. population lives in regions with suboptimal air quality (65). Moreover, non-white individuals residing in those regions will have more exposure to air pollutants relative to white and Native Americans (66). Bone loss may be significantly greater when considering the intersection of race, gender, and place of residence in studies examining the relationship between bone health and air pollution. Future studies could investigate whether these findings are generalizable to other populations and whether the inclusion of additional variables alters the effects observed in our study.

Our study had several strengths. Notably, we demonstrated associations using a relatively large sample size within a well-characterized, diverse cohort, ensuring adequate representation of participants across strata of SDOH measures. We analyzed bone loss at anatomical sites well-established as associated with pathological age-related conditions (67). To our knowledge, this is the first detailed study to examine the social determinants of vulnerability to NO_x−_ and SO_2_-related bone damage in a large population of postmenopausal women.

There are multiple limitations of our study that warrant consideration. Our samples consisted exclusively of older women, which limited the generalizability of the findings to younger women and men. Although false discovery rate (FDR) correction was not applied, associations were largely consistent across exposure periods, supporting the robustness of the observed trend. Nevertheless, these findings should be interpreted as suggestive, which may need further confirmation. Despite extensive adjustment for demographics, physical activity, smoking, diet modification (including calcium and vitamin D intake), and hormone therapy. We acknowledge that the study did not adjust for several important determinants of bone mineral density, including anti-resorptive medications (e.g., bisphosphonates), history of fractures, and family history of osteoporosis. While anti-resorptive medications may plausibly modify these associations, the available data does not provide sufficient evidence. An uneven distribution of these factors across socioeconomic strata could introduce residual confounding, thereby biasing associations with air pollution. However, we reason that excluding these covariates would not significantly alter the magnitude of the associations. In fact, a UK Biobank survival analysis showed that air pollution strongly predicted lower bone mineral density and increased osteoporosis risk (42). Indeed, the history of prior fracture reduces bone strength. We therefore ran a quick multivariable analysis, which suggested potential associations with air pollution and incident fractures. Further work is required to characterize these associations in greater detail. There is no doubt that including relevant variables would reduce residual variance and improve the precision of the estimates.

Numerous SDOH were unavailable or unassessed, including employment, social support, language, incarceration, healthcare access, health literacy, housing quality, transportation, and access to parks. We focused on key SDOH to provide an initial assessment of their role in vulnerability to air pollution-associated bone damage. Additionally, we lacked data on early-life SDOH (e.g., family socioeconomic status, childhood neighborhood characteristics, residential history). The geographic region was included as a proxy but offers limited spatial precision. Occupational histories, which could reflect long-term physical activity, SES, and toxic exposure, were also unavailable. We also did not have information on institutionalization or nursing home residency, despite its established link with fracture risk. Also, while BMD is a valuable surrogate of bone health, it is an intermediate outcome and less clinically definitive than fracture events. Although BMD is one of the most widely used tools for future fracture prevention, better tools are still needed. We have not adjusted for comorbid diseases and have included only a select few air pollutants to investigate these associations. The reason for not including comorbidities is that common clinical conditions, such as diabetes, that can increase the risk of fractures, have not been well characterized or available in WHI (e.g., chronic kidney disease). We did not seek to disaggregate the effects by race, partly because the non-white individuals were underrepresented in the dataset. Underrepresentation in the WHI may limit equity insights and is therefore underpowered to detect differences by race. However, analyses among non-Whites in other studies showed associations in the same direction. For example, Asians have a high fracture risk and conditions that aggravate bone loss (68). Finally, housing, an often-overlooked SDOH, may mediate the relationship between social disadvantage and NO_x_ and SO_2_-related poor health, particularly in women, but this was not included in the current study. A substandard housing environment exposes individuals to numerous stressors like environmental hazards, structural decay, overcrowding, etc., which may exacerbate psychological stress and social isolation (69).

Future studies should investigate the intersection of housing conditions, NO_x_ and SO_2_, and bone health, particularly in marginalized populations. Our findings build upon preceding research by quantifying the increased bone loss associated with cumulative exposure to NO_x_ and SO_2_ among less affluent individuals (70). Such quantitative evidence is vital for informing policymakers about the disproportionate burden on disadvantaged groups and advancing policy changes. Addressing social inequality and environmental injustice is key to building a healthier and more equitable society.

## 5 Conclusion

In conclusion, we found that social adversity may amplify NOx– related bone loss. The nSES index seemed to outperform other individual-level indices, and more consistent suggestive associations were observed for NOx than for SO_2_. Addressing social inequities may boost resilience against NOx–induced bone damage, reduce bone loss, and lower the risk of fractures among aging women.

## Data Availability

The raw data supporting the conclusions of this article will be made available by the authors, without undue reservation.
